# A Systematic Review and Meta-Analysis of Surgical Feasibility and Outcomes Following Neoadjuvant Immune Checkpoint Inhibition in Resectable Stage III and IV Melanoma

**DOI:** 10.1245/s10434-026-20067-8

**Published:** 2026-07-09

**Authors:** T. Read, G. Nair, G. Velli, M. David, G. Bayley, Wen Xu, A. Barbour, B. M. Smithers, V. Atkinson

**Affiliations:** 1 Princess Alexandra Hospital, Brisbane, Woolloongabba, Queensland Australia; 2 The University of Queensland, Faculty of Medicine, Southern Clinical Division, Princess Alexandra Hospital, Brisbane, Australia; 3 The Daffodil Centre, Faculty of Medicine and Health, University of Sydney, Sydney, Australia; 4 School of Medicine and Dentistry, Griffith University, Gold Coast, Australia; 5 Queensland Melanoma Institute, Brisbane, Australia

**Keywords:** Neoadjuvant, Immunotherapy, Metastatic melanoma, Surgery, Treatment outcomes

## Abstract

**Background:**

Neoadjuvant immune checkpoint inhibition (ICI) has demonstrated high pathological response rates in resectable stage III melanoma and is increasingly used for selected patients with low-volume stage IV disease. However, the surgical and perioperative implications remain incompletely defined. A systematic review and meta-analysis were performed to evaluate oncologic, surgical, and immune-related outcomes following neoadjuvant ICI and curative-intent surgery.

**Materials and Methods:**

MEDLINE, EMBASE, and the Cochrane Library, were systemically searched for both prospective and retrospective studies including adults with resectable stage III or oligometastatic stage IV melanoma treated with neoadjuvant ICI followed by planned surgery. Outcomes included pathological response, recurrence, failure or delay to surgery, perioperative complications, and treatment-related adverse events. Pooled estimates were calculated using random-effects meta-analyses.

**Results:**

A total of 20 studies comprising 1384 patients were included. The pooled proportions of pathological complete response were 0.33 (CI 0.26–0.40), major pathological response 0.46 (CI 0.38–0.54), and overall pathological response 0.59 (CI 0.51–0.67). Pathological nonresponse occurred in 0.30 (CI 0.25–0.36). Failure or delay to surgery occurred in 0.09 (CI 0.07–0.12), with disease progression during neoadjuvant therapy reported in 0.06 (CI 0.04–0.10). Major perioperative complications (Clavien–Dindo ≥ III) occurred in 0.08 (CI 0.05–0.13), while major treatment-related toxicities (CTCAE grade ≥ III) occurred in 0.25 (CI 0.18–0.34). Comparative analyses demonstrated similar perioperative complication rates between neoadjuvant and adjuvant approaches, with lower recurrence risk favoring neoadjuvant therapy (*p* < 0.01).

**Conclusions:**

Neoadjuvant ICI produces substantial pathological responses while preserving surgical feasibility in resectable stage III and selected stage IV melanoma. Operative morbidity appears acceptable; however, systemic toxicity is common, and perioperative reporting remains heterogeneous.

**Supplementary Information:**

The online version contains supplementary material available at https://doi.org/10.1245/s10434-026-20067-8.

Neoadjuvant immune checkpoint inhibition has emerged as an effective treatment strategy for patients with stage III melanoma and is increasingly applied to selected subgroups with low-volume, resectable stage IV disease.^1–3^ Randomized trials and prospective cohort studies have demonstrated high pathological response rates and encouraging survival outcomes with neoadjuvant PD-1–based therapy, particularly when combined with CTLA-4 blockade.^3–5^ Within this evolving treatment paradigm, surgical resection remains central to curative-intent management.^6–8^ This approach provides locoregional disease control and pathological assessment that informs response-adapted treatment strategies.^3,4,8–10^ However, while oncologic outcomes following neoadjuvant immune checkpoint inhibition (ICI) have been widely reported, the downstream surgical and perioperative implications of this approach remain incompletely defined.^11^

From a clinical perspective, uncertainty persists regarding the feasibility and optimal timing of surgery following neoadjuvant therapy, the incidence and severity of perioperative complications, and the impact of treatment-related toxicity on surgical decision-making and subsequent adjuvant therapy sequencing.^2,8,11,12^ Existing studies demonstrate substantial heterogeneity in neoadjuvant regimens, number of treatment cycles, timing to surgery, and extent of surgical resection, alongside inconsistent reporting of surgery-specific outcomes such as delays or inability to proceed to surgery, disease progression during the neoadjuvant phase, and postoperative morbidity.^3,10,11^ This limited integration of perioperative risk into response-adapted surgical strategies has constrained the ability to comprehensively counsel patients, optimise treatment sequencing, and interpret surgical outcomes alongside established, response-driven oncologic endpoints.^12^ As neoadjuvant immune checkpoint inhibitors become increasingly embedded in the routine management of locoregionally advanced, resectable melanoma, this has important implications for multidisciplinary decision-making and future trial design.^12,13^

This systematic review aims to comprehensively evaluate immune-related, surgical, and perioperative complications, alongside oncologic response and survival outcomes, in patients with resectable stage III and low-volume stage IV melanoma undergoing curative-intent surgery following neoadjuvant immunotherapy. By synthesising data from numerous high-quality studies, this review will provide an assessment of surgical feasibility, perioperative morbidity, and treatment sequencing in the neoadjuvant setting. It seeks to inform multidisciplinary decision-making, support response-adapted surgical strategies, and identify key evidence gaps to guide future studies incorporating surgery-specific endpoints.

## Methods

### Search Strategy

**Fig. 1:**
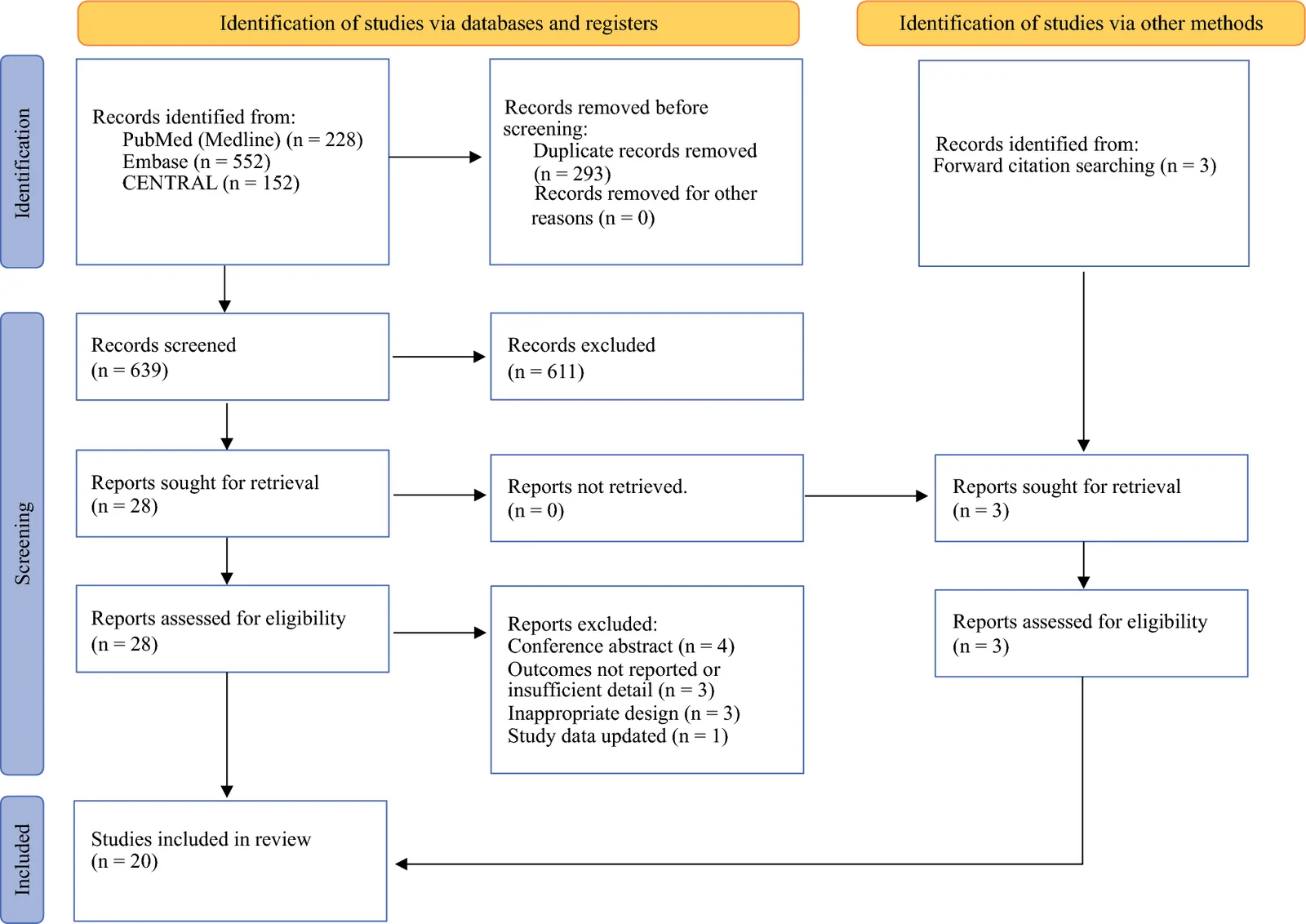
PRISMA flow diagram

The PRISMA guidelines were used to prepare this article in accordance with accepted research methodology (Fig. 1). ChatGPT4.0 was used for brainstorming terms and a text frequency analysis tool was applied to publications that met inclusion criteria to identify and refine keywords, synonyms, and subject indexing terms. Search filters were integrated into the search to exclude non-human, laboratory, cadaver and pediatric studies. The strategy was assessed using the Peer Review of Electronic Search Strategies (PRESS) checklist and validated through evaluation against publications already identified as meeting inclusion criteria.^14^ Key terms used in the search included “neoadjuvant,” “immunotherapy,” “melanoma,” “surgery,” and terms related to specific outcomes, and complications such as “adverse events,” “peri-operative,” and “survival” in combination with Boolean operators. The search was limited to English-language studies published since 1980.

Searches were conducted in the MEDLINE (PubMed), EMBASE, and the Cochrane Library databases, with additional algorithmic searches through Google and Google Scholar. Citation searching was conducted through forward citation searching of relevant bibliographies and manual citation review. The search was adapted for Clinical Knowledge Network (CKN) integrated search layer to identify further citations.^15^ The last search was completed on January 7, 2026. Searches were re-run prior to the final analysis to identify any further studies. Duplicate results were removed using EndNote. Following duplicate removal, titles and abstracts were independently screened by two reviewers against predefined inclusion and exclusion criteria. Full-text review was subsequently performed independently by the same reviewers to determine final eligibility. No AI-assisted screening or study selection tools were used. Search terms and search syntax in MEDLINE (PubMed) format and further adapted searches are included in the appendices. The Newcastle-Ottawa Scale was used to evaluate methodological quality and the risk of bias for non-randomized studies (See supplementary material 1). The study was prospectively registered on the Prospero Database (National Institute for Health and Care Research).

### Inclusion/Exclusion Criteria

Studies were considered appropriate for inclusion if they evaluated surgery-specific and oncological outcomes in patients with resectable AJCC stage III and low-volume stage IV melanoma undergoing curative-intent surgery following systemic neoadjuvant ICI. Patients over the age of 18 years were included. Studies primarily examining other neoadjuvant regimens that included targeted therapies (e.g. BRAF or MEK inhibitor agents) or locoregional intralesional agents such as T-VEC were excluded. Prospective and retrospective case series, cohort studies, and clinical trials were considered. Conference abstracts, case reports (*n* ≤ 5), editorial correspondence and other anecdotal reports were not included. Articles published in languages other than English were excluded. Minimum outcome data for inclusion were pathological response, event-free, recurrence-free or overall survival, pattern of recurrence, delays or inability to proceed with surgery, complication rates, and follow–up duration. A detailed description of patient selection and the treatment protocol, including the regimen and timing of immune checkpoint inhibitors relative to surgery was also necessary.

### Outcome Assessments

Outcome definitions and reporting varied across included studies. Surgical and perioperative outcomes were extracted from the results sections and, when necessary, supplementary materials, and recorded in tabulated or narrative form. Primary surgical outcomes included a delay or inability to proceed to surgery, time from the last dose of neoadjuvant treatment or perioperative complications. Where reported, complications were extracted according to the grading system used in the original study, most commonly the Common Terminology Criteria for Adverse Events (CTCAE)-defined immunotherapy and surgery-attributed adverse events (using the Clavien-Dindo grading system). Oncologic outcomes, including pathological response and survival endpoints, were extracted as reported by study authors. No attempt was made to reclassify outcomes across differing grading systems.

### Data Analysis

Two authors (TR and GN) cross-verified then independently reviewed extracted data from eligible studies. Where differences in interpretation occurred, these were addressed through consensus discussion and reconciled by the senior author. The statistical heterogeneity was determined for the primary outcomes using the Chi-square (Cochran’s Q) statistic and Higgins’ method of calculation with the respective I^2^ and *p*-values provided.^16,17^ For comparative analyses of neoadjuvant versus adjuvant strategies, pooled risk differences were estimated using random-effects meta-analysis with Restricted Maximum Likelihood (REML) estimation to account for between-study heterogeneity. Pooled estimates of proportions with corresponding 95% confidence intervals (CI) were calculated on the basis of the Freeman-Tukey double arcsine transformation within a random–effect model framework.^18,19^ The potential publication bias was assessed visually using the funnel plot and statistically using Egger’s test.^20^ Statistically significant differences were defined by a *p*-value < 0.05.

## Results

The initial search across all databases identified 932 publications, of which 293 were duplicates. All identified studies were published in English. Of the remaining 639 records screened, 611 did not meet inclusion criteria. Twenty-eight articles underwent full-text review, of which a further eleven were excluded. All included publications were assessed for subsequent notifications of errata, retractions, errors, or omissions. Three additional studies were identified through forward citation searching. In total, twenty publications met eligibility criteria and were included in the formal analysis. The PRISMA search strategy is summarised in Fig. 1. Included studies comprised phase I–III randomised trials, prospective cohorts, and case series.

Twenty studies reported on the use of neoadjuvant immune checkpoint inhibitors, administered as single-agent or dual-agent regimens or in combination with lesion-directed therapies (Table 1). Anti–PD-1 and CTLA-4 agents included pembrolizumab, nivolumab, ipilimumab, relatlimab, and toripalimab, as well as novel combinations such as tobemstomig, tiragolumab, and atezolizumab. After pooling data across studies, 1384 treated patients were available for analysis. Median event-free survival ranged from 11 to 36 months and follow-up duration ranged from 7 to 69 months.

**Table 1:** Neoadjuvant study details, response rates and survival outcomes^1,3–5,21–35^

Study	Study design	NHMRC Level of evidence	Neoadjuvant regimen	Sample size	Pathological complete response (pCR)	Major pathological response (MPR)	Pathological partial response (pPR)	Overall pathological response (ORR)	Pathological non-response (pNR)	Median relapse-free survival (months)	Median event-free survival (months)	Median overall survival (months)	Relapse / pattern of recurrence	Median Follow-up Duration (months)
Versluis et al.^10^ OpACIN- Neo	Phase II RCT	II	Ipilimumab + Nivolumab (three arms)	86	37 (43%)	52 (60%)	12 (14%)	64 (74%)	19 (22%)	NR 3-year RFS 82%	NR 3-year EFS 81%	NR 3-year OS 92%	14 (16%) 5 regional 9 distant	47
Versluis et al.^10^ OpACIN	Phase I RCT	II	Ipilimumab + Nivolumab	10	3 (30%)	6 (60%)	1 (10%)	7 (70%)	3 (30%)	NR 5-year RFS 70%	NR 5-year EFS 70%	NR 5-year OS 90%	4 (40%) 1 regional 3 distant	69
Patel et al.^1^ SWOG S1801	Phase II RCT	II	Pembrolizumab	154	28 (18%)	NR	NR	NR	NR	NR	NR 2-year EFS 72%	NR 3-year OS 80%	9 (6%) sites NR	15
Blank et al.^3^ NADINA	Phase III RCT	II	Ipilimumab + Nivolumab	212	100 (47%)	125 (59%)	17 (8%)	142 (67%)	55 (26%)	NR	NR 1-year EFS 84%	NR	23 (11%) sites NR	11
Reijers et al.^4^ PRADO	Phase II Cohort	III	Ipilimumab + Nivolumab	99	48 (49%)	60 (61%)	11 (11%)	71 (72%)	21 21%	NR 2-year RFS 85%	NR 2-year EFS 80%	NR 2-year OS 95%	22 (22%) sites NR	28
Dummer et al.^21^ KEYMAKER-UO2	Phase I/II RCT	II	Pembrolizumab (three arms)	66	23 (35%)	30 (45%)	15 (23%)	45 (68%)	16 (24%)	NR	NR	NR	4 (6%) sites NR	26
Shafique et al.^22^	Phase Ib Case Series	IV	Pembrolizumab	51	7 (14%)	10 (20%)	NR	10 (20%)	NR	NR	NR	NR 5-year OS 100% vs 76%	23 (45%) 11 regional 9 distant 3 both	63
Song et al.^23^	Cohort	III	Pembrolizumab, Nivolumab, Ipilimumab or Ipilimumab + Nivolumab	41	5 (12%)	5 (12%)	23 (56%)	28 (72%)	11 (28%)	NR	NR 3-year DFS 62%	NR 3-year MSS 78%	9 (22%) sites NR	26
Tarhini et al.^24^	Phase I RCT	II	Ipilimumab (two arms)	30	9 (32%)	9 (32%)	2 (7%)	11 (39%)	NR	NR	1-year PFS 86%	NR 2-year OS 89%	11 (39%) sites NR	32
Tarhini et al.^25^	Case Series	IV	Ipilimumab	35	NR	NR	NR	NR	NR	NR	11 months (PFS) 1-year PFS 47%	NR 1-year OS 97A%	6 (17%) Sites NR	16
Amaria et al. 2022	Case Series	IV	Nivolumab + relatlimab	30	17 (57%)	19 (63%)	2 (7%)	21 (70%)	8 (27%)	NR 2-year RFS 82%	NR 2-year EFS 81%	NR 2-year OS 88%	3 (10%) 2 regional 1 distant	24
Zijlker et al.^27^	Cohort	III	Ipilimumab + Nivolumab	44	NR	23 (52%)	6 (14%)	29 (66%)	15 (34%)	NR	NR	NR	NR	NR
Liu et al.^28^	Phase Ib Case Series	IV	Toripalimab	30	4 (13%)	9 (30%)	12 (40%)	21 (70%)	6 (20%)	NR 2-year RFS 82%	NR 2-year EFS ~75%^#^	NR	5 (17%) 3 regional 2 distant	36
Najjar et al.^29^	Phase Ib/II Case Series	IV	Pembrolizumab	30	13 (43%)	17 (57%)	0	17 (57%)	13 (43%)	2-year RFS 70%^#^	NR	NR 2-year OS 83%^#^	5 (17%) Sites NR	38
Nelson et al.^30^ NEO-MEL	Cohort	III	Nivolumab +/- Ipilimumab	118	41 (35%)	49 (42%)	11 (9%)	60 (51%)	36 (31%)	1-year RFS 74%	1-year EFS 72%	NR	28 (24%) Sites NR	12
Long et al.^31^ Morpheus	Phase Ib/II RCT	II	Tobemstomig or Tobemstomig + tiragolumab or Atezolizumab + tiragolumab or Ipilimumab-Nivolumab	102	47 (46%)	57 (56%)	13 (13%)	70 (69%)	24 (24%)	NR	NR	NR	NR	11-15
Tomsitz et al.^32^	Case Series	IV	Ipilimumab + nivolumab	24	8 (33%)	12 (50%)	1 (4%)	13 (54%)	11 (46%)	NR	NR	NR	6 (25%) Sites NR	22
Hou et al.^33^	Cohort	III	Pembrolizumab or toripalam + interferon- -α1b	32	6 (19%)	NR	4 (13%)	10 (32%)	22 (69%)	13 2-year RFS 40%	NR	NR	NR	19
Tarhini et al.^34^	Phase II Case Series	IV	Tavokinogene telseplasmid + nivolumab	16	9 (60%)	12 (80%)	NR	NR	1 (6%)	NR 1-year RFS 93%	NR 1-year EFS 88%	NR 1-year OS 94%	NR	15
Nardin et al.^35^ NEOMEL	Cohort	III	Anti-PD-1 monotherapy or Ipilimumab + nivolumab	174	50 (29%)	59 (34%)	14 (8%)	73 (42%)	38 (22%)	29 1-year RFS 71%	36 1-year EFS 73%	NR	27 (16%) 17 local/regional 10 distant	7
Non-weighted Totals	**NA**	**NA**	**NA**	**1384**	**455 (33%)**	**554 (40%)**	**144 (10%)**	**692 (50%)**	**299 (22%)**	**NA**	**NA**	**NA**	**199 (14%)**	**7 - 69**

*RCT* Randomized Control Trial, *NR* Not Reported, *NA* Not Applicable ^#^Derived from published KM survival curve estimates

Studies included patients with resectable stage IIIB–D disease or oligometastatic stage IV melanoma in whom definitive surgery was planned and incorporated acral melanoma and in-transit metastases (Table 2). The planned interval between the final neoadjuvant treatment cycle and surgery was typically 3–5 weeks. Definitive surgery consisted of therapeutic lymphadenectomy with or without metastectomy in all studies except one, which specifically examined index lymphadenectomy followed by response-adapted completion lymphadenectomy.

**Table 2:** Neoadjuvant surgical and perioperative outcomes^1,3–5,21–35^

Study	Disease substages III (A/B/C/D) or IV	Regimen	Type or extent of nodal surgery	Time from last neoadjuvant therapy to surgery	Delay or inability to undergo surgery	Disease progression during neoadjuvant treatment	Perioperative complication rate (Clavien–Dindo ≥ III)	Major complications CTCAE Grade ≥I 3	Predictive biomarkers
Versluis et al.^10^ OpACIN Neo	Stage III	2 × neoadj	Therapeutic lymphadenectomy	3 weeks	0	2	NR	44 (46%)	N
Versluis et al.^10^ OpACIN	Stage III	2 × neoadj 2 × adjuvant	Therapeutic lymphadenectomy	3 weeks	NA	NA	NA	NA	N
Patel et al.^1^ SWOG S1801^	Stage IIIB-D or IV	3 × neoadj 15 × adj	Therapeutic lymphadenectomy +/- metastectomy	≤ 5 weeks	14	12	9 (6%)	25 (16%)	N
Blank et al.^3^ NADINA	Stage III	2 × neoadj +/− Up to 46 weeks adj	Therapeutic lymphadenectomy	3 weeks	13	5	28 (13%)	64 (30%)	N
Reijers et al.^4^ PRADO	Stage IIIB-D	2 × neoadj Up to 12 months adj	Index lymphadenectomy +/- completion lymphadenectomy (TLND) response	3 weeks	5	2	2 (2%)	30 (30%)	N
Dummer et al.^21^ KEYMAKER-UO2	Stage IIIB-D	1-2 × neoadj Up to 12 months adj	Therapeutic lymphadenectomy	3–6 weeks	4	4	NR	10 (15%)	Y
Shafique et al.^22^	Stage III or IV	1 × neoad	Therapeutic lymphadenectomy +/− metastectomy	3 weeks	0	0	0	8 (17%)	N
Song et al.^23^	Stage IIIB-D	1 × neoadj	Therapeutic lymphadenectomy	≤ 3 weeks	0	0	NR	4 (10%)	N
Tarhini et al.^24^	Stage III	2 × neoadj Up to 46 weeks adj	Therapeutic lymphadenectomy	3–5 weeks	**0**	9	NR	25 (83%)	Y
Tarhini et al.^25^	Stage III	2 × neoadj 2 × adj	Therapeutic lymphadenectomy	3–5 weeks	0	8	NR	14 (40%)	Y
Amaria et al. 2022	Stage III or IV	2 × neoadj 10 × adj Up to 46 weeks	Therapeutic lymphadenectomy +/− metastectomy	5 weeks	1	1	NR	8 (26%)	Y
Zijlker et al.^27^	Stage III	2 × neoadj	Therapeutic lymphadenectomy	3 weeks	0	0	7 (16%)	26 (59%)	N
Liu et al.^28^	Stage III or IV	4-6 × neoadj Up to 12 months adj	Therapeutic lymphadenectomy +/− metastectomy	2–3 weeks	3	3	NR	4 (13%)	Y
Najjar et al.^29^	Stage III or IV	2 × neoadj Up to 46 weeks adj	Therapeutic lymphadenectomy +/−metastectomy	2–3 weeks	0	1	NR	≥13 (43%)	Y
Nelson et al.^30^ NEO-MEL	Stage III or IV	2 × neoadj Up to 12 × adj	Index nodectomy or therapeutic lymphadenectomy +/− metastectomy	3–5 weeks	14	11	3 (3%)	11 (9%)	N
Long et al.^31^ Morpheus	Stage III	2 × neoadj	Therapeutic lymphadenectomy	2 weeks	13	3	NR	21 (21%)	Y
Tomsitz et al.^32^	Stage III or IV	2 × neoadj +/- 2× adj	Therapeutic lymphadenectomy +/− metastectomy	3 weeks	4	0	5 (21%)	3 (13%)	N
Hou et al.^33^	Stage III or IV	1 × neoadj (pembro or toripalamib) + 3 × interferon- -α1b > 3 × adj	Therapeutic lymphadenectomy	4 weeks	4	NR	4 (13%)	11 (34%)	N
Tarhini et al.^34^	Stage III or IV	3 × neoadj Up to 9 × adj	Not specified	2–4 weeks	1	1	NR	5 (31%)	Y
Nardin et al.^35^ NEOMEL	Stage III or IV	2–3 × neoadj +/− adj	Therapeutic lymphadenectomy +/− metastectomy	8 weeks	25	10	10 (6%)	15 (9%)	N
Non-weighted Totals	**NA**	**NA**	**NA**	**NA**	**101 (7%)**	**72 (5%)**	**68 (7%)**	**341 (25%)**	**NA**

*NR* Not Reported*, NA* Not Applicable

In subgroup analyses, five comparative studies evaluated both neoadjuvant and adjuvant treatment strategies (Table 3). After pooling data, 424 patients treated with an adjuvant approach and 533 patients treated with a neoadjuvant approach were available for comparison. Non-weighted perioperative complication rates ranged from 4–15% in the adjuvant groups and 6–13% in the neoadjuvant groups (non-weighted mean 10% in both). Rates of major systemic treatment-related complications (CTCAE grade ≥III) ranged from 11–90% in the adjuvant cohorts and 10–46% in the neoadjuvant cohorts (non-weighted means 16% vs 28%, respectively). Mean follow-up duration across comparative studies ranged from 10 to 47 months.

**Table 3:**
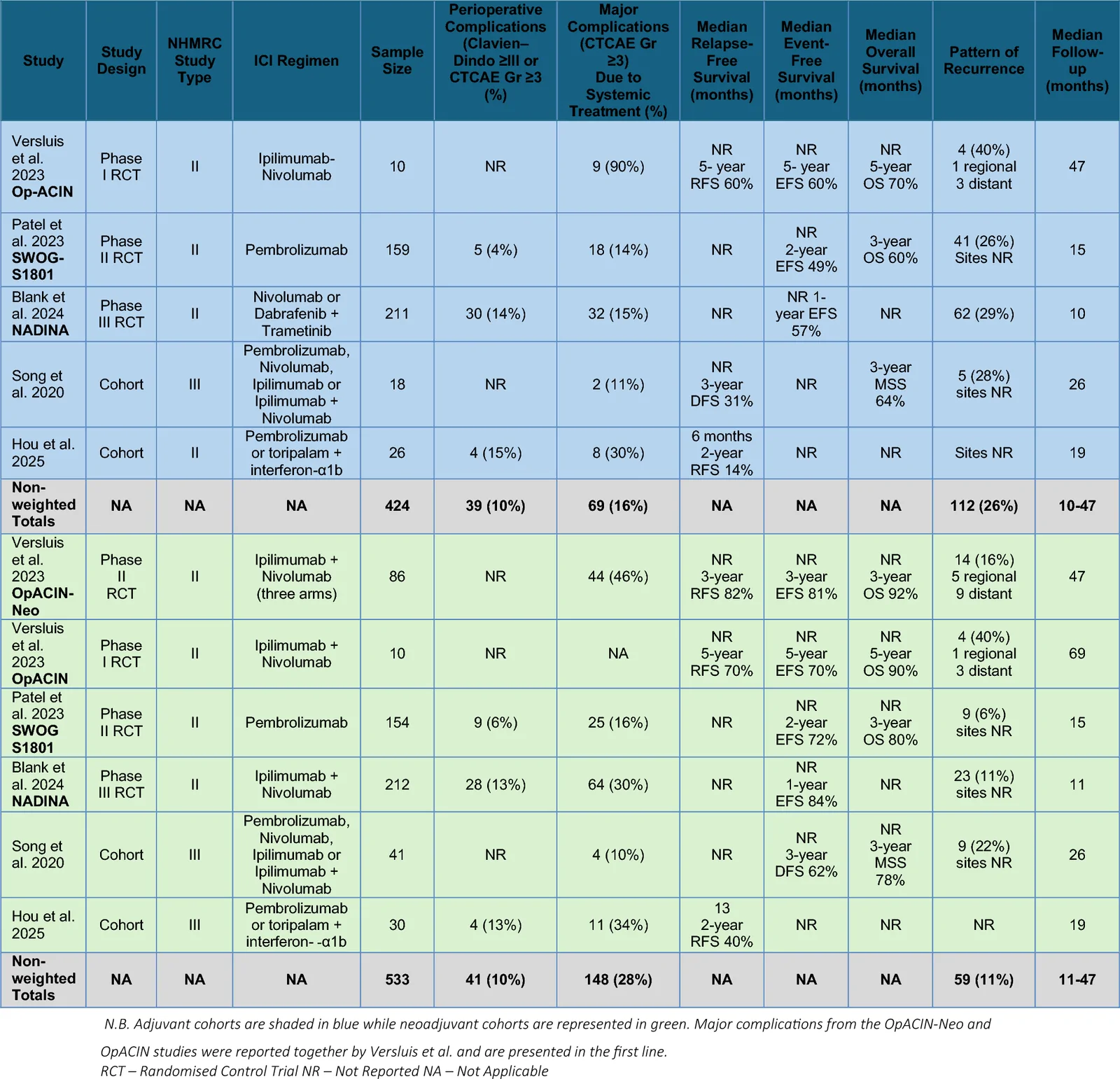
Cohort studies describing response rates, survival outcomes, surgical and perioperative complications for comparative adjuvant and neoadjuvant subgroups^1,3,5,23,33^

A random-effects meta-analyses using a REML approach were performed and identified the following differences in the pooled data (See supplementary material 2). Seventeen studies were included in the pooled analyses of pathological response outcomes. The pooled proportion of pathological complete response (pCR) was 0.33 (95% CI 0.26–0.40). Major pathological response (MPR) occurred in 0.46 (95% CI 0.38–0.54), pathological partial response (pPR) in 0.15 (95% CI 0.10–0.23). The overall pathological response (OPR = pCR + pPR) rate was 0.59 (95% CI 0.51–0.67), indicating measurable tumour regression in approximately 60% of patients. Pathological non-response (pNR), reported in 14 studies, occurred in 0.30 (95% CI 0.25–0.36). Heterogeneity was moderate to high across response endpoints (I^2^ = 63.9–84.8%). No statistical evidence of publication bias was detected for pathological response outcomes. Across 16 studies evaluating recurrence, the pooled recurrence proportion was 0.19 (95% CI 0.14–0.25), with high heterogeneity (I^2^ = 77.4%). Relapse-free survival (RFS), reported in eight studies, demonstrated a pooled proportion of 0.74 (95% CI 0.65–0.81). Median relapse-free survival, event-free survival, and overall survival were not pooled because of substantial variation in follow-up duration and reporting methods.

**Fig. 2:**
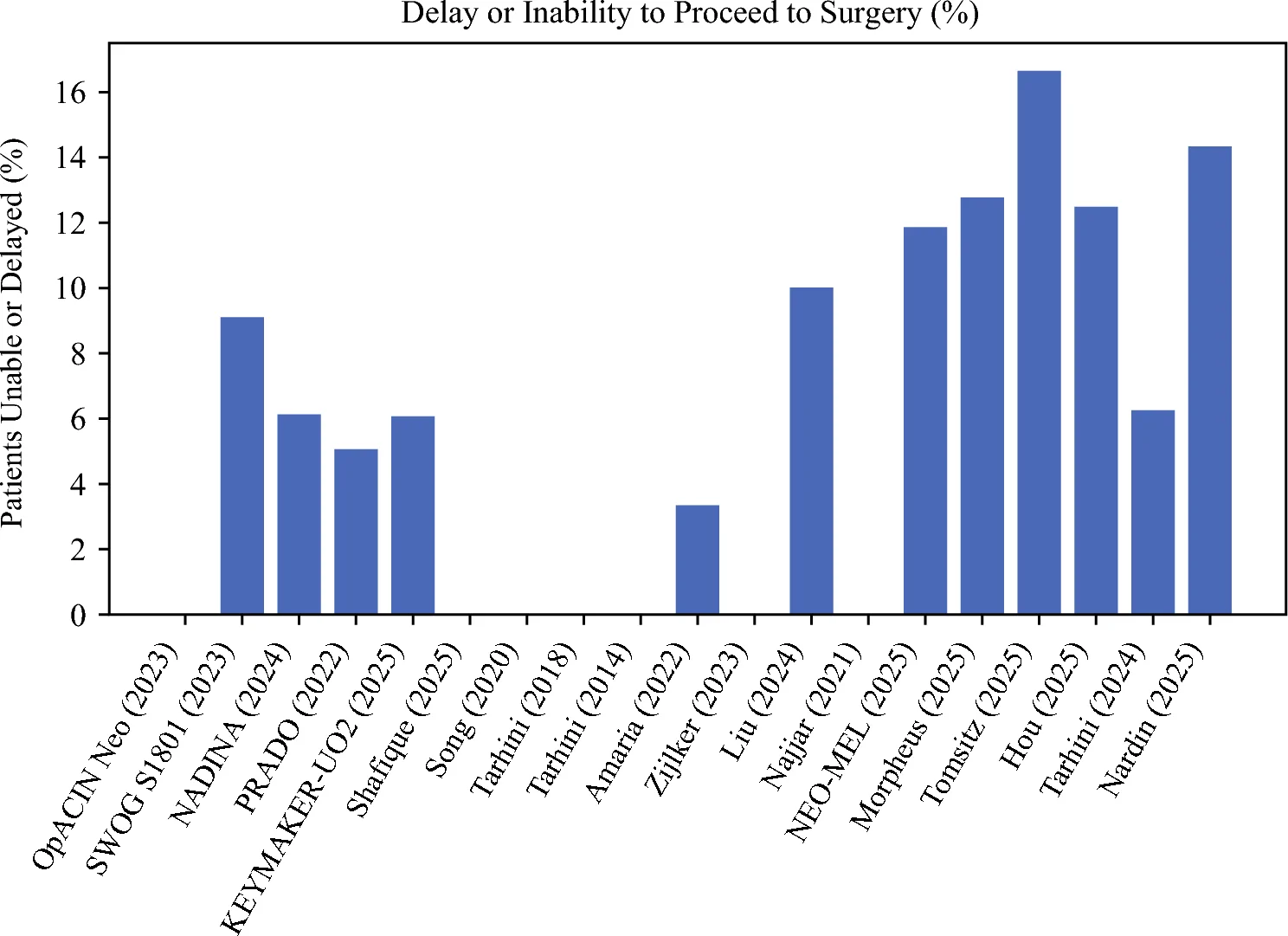
Surgical feasibility—the proportion of patients unable to proceed to planned curative-intent surgery or experiencing clinically significant delay following neoadjuvant immune checkpoint inhibition across contemporary trials. Reasons for delay or nonprogression most frequently included disease progression and immune-related adverse events (irAEs)

Nineteen studies reported delay or inability to proceed to definitive surgery (Fig. 2), with a pooled proportion of 0.09 (95% CI 0.07–0.12) and moderate heterogeneity (I^2^ = 40.6%). Disease progression during neoadjuvant therapy, reported in 18 studies, occurred in 0.06 (95% CI 0.04–0.10), with high heterogeneity (I^2^ = 69%); Egger’s test suggested small-study effects (p = 0.01). Major perioperative complications (Clavien–Dindo ≥ III), reported in nine studies, occurred in 0.08 (95% CI 0.05–0.13), with high heterogeneity (I^2^ = 71.3%); publication bias was not assessed given the limited number of studies. Major treatment-related complications (CTCAE grade ≥III), reported in 19 studies, occurred in 0.25 (95% CI 0.18–0.34), with high heterogeneity (I^2^ = 88.3%); no evidence of publication bias was detected (*p* = 0.62).

In comparative meta-analysis of perioperative complications, the adjuvant group demonstrated a marginally higher risk than the neoadjuvant group; however, this difference was not statistically significant (risk difference [RD] 0.01; 95% CI -0.05 to 0.02; *p* = 0.48). For major complications, the adjuvant group had a numerically lower risk compared with the neoadjuvant group, and this difference did reach statistical significance (RD -0.07; 95% CI -0.14 to -0.01; *p* = 0.04). Patterns of recurrence differed between strategies. The adjuvant group demonstrated a significantly higher risk of recurrence compared with the neoadjuvant group (RD 0.18; 95% CI 0.13 to 0.24; *p* < 0.01), favouring the neoadjuvant approach with low statistical heterogeneity (I^2^ < 1%).

## Discussion

This systematic review synthesises contemporary evidence regarding the surgical implications of neoadjuvant immune checkpoint inhibition in resectable stage IIIB–D and selected oligometastatic stage IV melanoma. Across 20 studies including 1,384 patients, neoadjuvant therapy demonstrated substantial pathological activity, with 33% achieving pathological complete response and 40% achieving major pathological response. Overall pathological response was observed in 50% of patients, and recurrence occurred in 14% at follow-up ranging from 7 to 69 months. These findings indicate meaningful oncologic activity in the preoperative setting and support the biological rationale for neoadjuvant immune checkpoint inhibition. However, approximately one-third of patients exhibited pathological non-response, highlighting the remaining unmet need of a clinically relevant treatment resistant subgroup. While deep pathological response has emerged as a promising early surrogate for durable disease control, its validity as a long-term predictor remains incompletely defined. These data support the efficacy of neoadjuvant ICI while emphasizing the need for improved biomarker-driven patient selection.

A central clinical concern is whether neoadjuvant therapy compromises safe progression to definitive surgery. Within this meta-analysis, surgery (definitive resection) remained achievable in the majority of patients. Across studies, surgery was typically performed within 3–5 weeks of the final treatment cycle, suggesting a consistent operative window. Disease progression during neoadjuvant therapy occurred in 6%, and 9% of patients experienced delay or inability to proceed to surgery. Major perioperative morbidity (Clavien–Dindo ≥III) was reported in 8%, whereas grade ≥III systemic treatment-related toxicities occurred in 25%. Chronic irAEs and permanent toxicity were infrequently reported, likely reflecting restricted adverse event reporting windows.^3,4^ Persistent immune-related toxicity has previously been described in 43–46% of patients treated with anti–PD-1 therapy for melanoma, with most events being mild (CTCAE grade I–II) and up to 37% resolving with extended follow-up.^37,38^ These data underscore the potential value of dose de-escalation strategies to mitigate systemic side-effects in appropriately selected patients following definitive surgery. In comparative analyses, perioperative complication rates were similar between neoadjuvant and adjuvant approaches, while systemic toxicity was numerically higher with neoadjuvant therapy. Collectively, these findings indicate that neoadjuvant ICI does not appear to substantially increase major surgical morbidity.

Importantly, interpretation of operative risk is constrained by heterogeneity in outcome definitions and reporting practices. Many studies applied CTCAE criteria, which primarily capture systemic toxicity, whereas fewer consistently reported surgery-specific complications using Clavien–Dindo grading. Data regarding return to theatre, wound complications, lymphoedema, and length of stay were inconsistently described. As a result, reported morbidity may reflect immunotherapy surveillance rather than standardised surgical outcome assessment, limiting cross-study comparability and precise integration of operative risk into treatment algorithms.

High pathological response rates have prompted interest in response-adapted surgical de-escalation. Trials such as PRADO and OpACIN-Neo demonstrate that major pathological response correlates with favorable event-free survival and may permit a reduction of nodal surgery in selected patients^.^^4,5^ However, these strategies were protocol-driven and implemented in carefully curated populations. Pathological complete response does not eliminate surgical risk, and mature regional control data remain limited. Extrapolation of de-escalation strategies beyond controlled settings appears worthwhile to minimise morbidity, however should be undertaken cautiously.

The most direct comparative surgical analysis, reported by Zijlker et al., found no increase in postoperative complications, operative duration, or failure to achieve a composite “textbook outcome” following neoadjuvant PD-1/CTLA-4 therapy compared with upfront lymphadenectomy.^27^ Although immune-related adverse events and corticosteroid exposure were common preoperatively, these did not translate into increased postoperative morbidity in the analyzed cohort. Nonetheless, the single-center design, modest sample size, restriction to combination blockade, and exclusion of patients unable to proceed to surgery limit generalizability. These findings reinforce the need for standardised, surgery-focused endpoints in prospective studies.

The interface between surgery and systemic therapy also warrants consideration. Although 7% of patients experienced delay or inability to proceed to surgery, the downstream effect on adjuvant therapy delivery is incompletely reported. Registry data from Rhodin et al. suggest that initiating adjuvant ICI within 6 weeks of surgery was not associated with improved outcomes and was associated with inferior recurrence-free survival.^36^ Delayed initiation did not compromise oncologic outcomes, supporting prioritization of postoperative recovery when clinically indicated. These observations have implications for interpreting survival endpoints across neoadjuvant and adjuvant paradigms and underscore the importance of coordinated surgical-medical reporting.

The inability to proceed to planned surgery should not uniformly be interpreted as treatment failure attributable to neoadjuvant therapy itself. Importantly, the subgroup of patients who develop locally unresectable disease requires further characterization. In some patients, interval development of disseminated metastatic disease may instead reflect underlying aggressive tumor biology that would likely have manifested irrespective of treatment sequencing. In this context, neoadjuvant therapy may function as a biologic selection tool, identifying patients less likely to derive durable benefit from surgical intervention due to occult systemic disease. Future studies should differentiate progression to unresectable locoregional disease directly precluding surgery from interval development of distant metastatic disease, which may instead alter the indication for or anticipated benefit of surgical intervention.

Furthermore, timing-related outcomes, including delays to surgery, may have been incompletely captured in retrospective real-world datasets, where documentation granularity and reporting consistency are inherently variable. Interpretation of interval radiologic progression also remains complex in the setting of ICI therapy, particularly given the recognized potential for pseudoprogression and delayed treatment responses. Reporting of progression assessment and subsequent management was heterogeneous across included studies, and confirmatory imaging protocols or standardized criteria distinguishing true progression from treatment-related inflammatory change were inconsistently described.

Most studies enrolled patients with stage IIIB–D disease and selected oligometastatic stage IV melanoma, including acral and in-transit presentations. Surgical feasibility appeared comparable across these groups, supporting inclusion of carefully selected stage IV patients in neoadjuvant strategies. However, survival outcomes were heterogeneous, and stage-specific surgical reporting was limited. Given the biological and prognostic differences between stage III and IV disease, future studies should provide granular, primary histological subtype and stage-stratified analyses to refine patient selection and multidisciplinary decision-making.

This review has several strengths, including comprehensive methodology, inclusion of randomised trials and contemporary cohorts, and prioritization of surgical endpoints alongside oncologic outcomes. Limitations include heterogeneity of ICI regimens, variation in surgical approaches, inconsistent complication grading, limited reporting of surgery-specific adverse events, and potential reporting bias. Statistical heterogeneity was high across several pooled endpoints, likely reflecting differences in staging, pathological definitions, treatment intensity, and follow-up duration. These differences likely reflect interinstitutional variation in treatment strategy however, they provided insight into real-world practice patterns and outcomes. Time-to-event outcomes could not be synthesised using uniform survival methods, limiting definitive conclusions regarding long-term benefit. These factors restrict the precision of pooled estimates but also reflect variation in real-world practice.

Results support the safe integration of neoadjuvant ICI into curative-intent strategies when delivered within a coordinated multidisciplinary framework, highlighting the essential partnership between oncology and surgical teams. Neoadjuvant immune checkpoint inhibition in resectable stage III and selected stage IV melanoma is associated with substantial pathological response and preserved surgical feasibility. The rate of major operative morbidity appears low and comparable to adjuvant strategies, although systemic toxicity remains frequent. Given the significant reduction in subsequent relapse and the durability of event-free survival observed in this study, the higher rate of systemic treatment-related adverse events associated with neoadjuvant therapy may be justified. As neoadjuvant approaches become more widely adopted, standardised reporting of delay to surgery and their causes, clearly attributed perioperative events, and surgery-specific endpoints will be essential to more precisely characterise operative risk and downstream benefits. Prospective trials incorporating surgical endpoints and biomarker-driven strategies, alongside registry-based analyses of long-term outcomes, will be important for better defining the role of surgery within the evolving paradigm of neoadjuvant immune checkpoint inhibition in contemporary melanoma care.

## Supplementary Information

Below is the link to the electronic supplementary material.

Supplementary file1 (DOCX 23 KB)

Supplementary file2 (DOCX 277 KB)

## Data Availability

The data that support the findings of this study are available on request from the corresponding author.
