# Supplementary material for: A Systematic Review and Meta-Analysis of Surgical Feasibility and Outcomes Following Neoadjuvant Immune Checkpoint Inhibition in Resectable Stage III and IV Melanoma
Source: Ann Surg Oncol. 2026 Jul 9;33(12):10772–84. doi: 10.1245/s10434-026-20067-8 (PMC13675365; doi:10.1245/s10434-026-20067-8)
Supplement: Supplementary file 1 [file 10434_2026_20067_MOESM1_ESM.docx]

| Study  **Supplementary Table 1: Newcastle-Ottawa Assessment Scale for Non-Randomised Studies** | Selection (Max 4) | | | | Comparability (Max 2) | | | Outcome (Max 3) | | | NOS Score (Good, fair, poor) |
| --- | --- | --- | --- | --- | --- | --- | --- | --- | --- | --- | --- |
|  | Representativeness of the exposed cohort | Selection of the non-exposed cohort | Ascertainment of exposure | Demonstration that outcome of interest was not present at start of study | The study controls for age, sex and marital status (one star) | Study controls for other factors (one star) | Cohorts are not comparable on the basis of the design or analysis controlled for confounders | Assessment of outcome | Was follow-up long enough for outcomes to occur | Adequacy of follow-up of cohorts |  |
| *Reijers et al. 2022* | X |  | X | X |  |  |  | X | X | x | Fair |
| *Shafique et al. 2025* | X |  | X | X | X | X |  | X | X | X | Good |
| *Song et al. 2020* | X | X | X | X | X | X |  | X | X |  | Good |
| *Tarhini et al. 2014* | X |  | X | X | X | X | X | X | X | X | Good |
| *Amaria et al. 2022* | X |  | X | X |  |  | X | X | X | X | Fair |
| *Ziiljker et al. 2023* | X | X | X | X | X | X | X | X | X | X | Good |
| *Liu et al. 2024* | X |  | X | X | X | X | X | X | X |  | Good |
| *Najjar et al. 2021* | X |  | X | X |  |  |  | X | X |  | Fair |
| *Hou et al. 2025* | X | X | X | X | X | X |  | X | X | X | Good |
| *Nardin et al. 2025* | X | X | X | X |  |  | X | X | X | X | Fair |
| *Nelson et al. 2025* | X |  | X | X |  |  | X | X | X | X | Fair |
| *Tomsitz et al. 2025* | X |  | X | X |  |  | X | X | X | X | Fair |
| *Tarhini et al. 2024* | X |  | X | X |  |  | X | X | X | X | Fair |
