# Supplementary material for: A Systematic Review and Meta-Analysis of Surgical Feasibility and Outcomes Following Neoadjuvant Immune Checkpoint Inhibition in Resectable Stage III and IV Melanoma
Source: Ann Surg Oncol. 2026 Jul 9;33(12):10772–84. doi: 10.1245/s10434-026-20067-8 (PMC13675365; doi:10.1245/s10434-026-20067-8)
Supplement: Supplementary file 2 [file 10434_2026_20067_MOESM2_ESM.docx]

**Outcome: PCR**

**Figure 3**

**Outcome: PCR (Continued)**

**Figure 4**

**Comments**

- A random effects meta-analysis with a Restricted Maximum Likelihood (REML) approach were used to pool proportions from 17 studies.
- The degree of statistical heterogeneity ($I^{2}=80.80\%)$between the 17 studies was found to be high (Higgins, 2003).
- The pooled proportion of Pathological Complete Responses was 0.33 (95%CI: 0.26 – 0.40).
- There was no evidence of publication bias with Egger’s test being non-significant (p-value=0.17) and no substantial asymmetry being displayed by the funnel plot.

**Outcome: MPR**

**Figure 5**

**Outcome: MPR (Continued)**

**Figure 6**

**Comments**

- A random effects meta-analysis with a Restricted Maximum Likelihood (REML) approach were used to pool proportions from 17 studies.
- The degree of statistical heterogeneity ($I^{2}=84.08\%)$between the 17 studies was found to be high (Higgins, 2003).
- The pooled proportion of Major Pathological Responses was 0.46 (95%CI: 0.38 – 0.54).
- There was no evidence of publication bias with Egger’s test being non-significant (p-value=0.77) and no substantial asymmetry being displayed by the funnel plot.

**Outcome: PPR**

**Figure 7**

**Outcome: PPR (Continued)**

**Figure 8**

**Comments**

- A random effects meta-analysis with a Restricted Maximum Likelihood (REML) approach were used to pool proportions from 16 studies.
- The degree of statistical heterogeneity ($I^{2}=84.12\%)$between the 16 studies was found to be high (Higgins, 2003).
- The pooled proportion of Pathological Partial Responses was 0.15 (95%CI: 0.10 – 0.23).
- There was no evidence of publication bias with Egger’s test being non-significant (p-value=0.16) and no substantial asymmetry being displayed by the funnel plot.

**Outcome: OPR**

**Figure 9**

**Outcome: OPR (Continued)**

**Figure 10**

**Comments**

- A random effects meta-analysis with a Restricted Maximum Likelihood (REML) approach were used to pool proportions from 17 studies.
- The degree of statistical heterogeneity ($I^{2}=84.79\%)$between the 17 studies was found to be high (Higgins, 2003).
- The pooled proportion of Overall Pathological Responses was 0.59 (95%CI: 0.51 – 0.67).
- There was no evidence of publication bias with Egger’s test being non-significant (p-value=0.84) and no substantial asymmetry being displayed by the funnel plot.

**Outcome: PNR**

**Figure 11**

**Outcome: PNR (Continued)**

**Figure 12**

**Comments**

- A random effects meta-analysis with a Restricted Maximum Likelihood (REML) approach were used to pool proportions from 14 studies.
- The degree of statistical heterogeneity ($I^{2}=63.90\%)$between the 14 studies was found to be moderate (Higgins, 2003).
- The pooled proportion of Pathological Non-responses was 0.30 (95%CI: 0.25 – 0.36).
- There was no evidence of publication bias with Egger’s test being non-significant (p-value=0.11) and no substantial asymmetry being displayed by the funnel plot.

**Outcome: Recurrence**

**Figure 13**

**Outcome: Recurrence (Continued)**

**Figure 14**

**Comments**

- A random effects meta-analysis with a Restricted Maximum Likelihood (REML) approach were used to pool proportions from 16 studies.
- The degree of statistical heterogeneity ($I^{2}=77.37\%)$between the 14 studies was found to be high (Higgins, 2003).
- The pooled proportion of Pathological Non-responses was 0.19 (95%CI: 0.14 – 0.25).
- There was no evidence of publication bias with Egger’s test being non-significant (p-value=0.82) and no substantial asymmetry being displayed by the funnel plot.

**Outcome: Delay or Inability to Undergo Surgery**

**Figure 15**

**Outcome: Delay or Inability to Undergo Surgery (Continued)**

**Figure 16**

**Comments**

- A random effects meta-analysis with a Restricted Maximum Likelihood (REML) approach were used to pool proportions from 19 studies.
- The degree of statistical heterogeneity ($I^{2}=80.80\%)$between the 17 studies was found to be low to moderate (Higgins, 2003).
- The pooled proportion of Delay or Inability to Undergo Surgery was 0.09 (95%CI: 0.07 – 0.12).
- There was evidence of publication bias with Egger’s test indicating statistical significance (p-value < 0.01) and substantial asymmetry as shown by a funnel plot, with 13 of the study’s 19 data points appearing on the left side of the reference line the funnel plot.

**Outcome: Disease Progression during Neoadjuvant Treatment**

**Figure 17**

**Outcome: Disease Progression during Neoadjuvant Treatment (Continued)**

**Figure 18**

**Comments**

- A random effects meta-analysis with a Restricted Maximum Likelihood (REML) approach were used to pool proportions from 18 studies.
- The degree of statistical heterogeneity ($I^{2}=69.01\%)$between the 18 studies was found to be moderate to high (Higgins, 2003).
- The pooled proportion of Disease Progression during Neoadjuvant Treatment was 0.06 (95%CI: 0.04 – 0.10).
- There was evidence of publication bias with Egger’s test indicating statistical significance (p-value = 0.01) and substantial asymmetry as shown by a funnel plot, with 13 of the study’s 18 data points appearing on the left side of the reference line the funnel plot.

**Outcome: Perioperative Complications**

**Figure 19**

**Comments**

- A random effects meta-analysis with a Restricted Maximum Likelihood (REML) approach were used to pool proportions from 9 studies.
- The degree of statistical heterogeneity ($I^{2}=71.27\%)$between the 9 studies was found to be moderate to high (Higgins, 2003).
- As less than 10 studies were included in the meta-analysis, no assessment of publication bias was undertaken as recommended by Chandler et al. (2019)

**Outcome: Major Complications**

**Figure 20**

**Outcome: Major Complications (Continued)**

**Figure 21**

**Comments**

- A random effects meta-analysis with a Restricted Maximum Likelihood (REML) approach were used to pool proportions from 19 studies.
- The degree of statistical heterogeneity ($I^{2}=88.34\%)$between the 19 studies was found to be high (Higgins, 2003).
- The pooled proportion of Major Complications was 0.25 (95%CI: 0.18 – 0.34).
- There was no evidence of publication bias with Egger’s test being non-significant (p-value=0.62) and no substantial asymmetry being displayed by the funnel plot.

**Outcome: Comparative Perioperative Complications**

**Figure 22**

**Outcome: Perioperative Complication**

**Comments**

- Overall, the Adjuvant group had a lower risk than the Neoadjuvant group in developing a Perioperative Complication but this risk difference was not statistically significant i.e. RD = -0.01 (95% CI: -0.05 to 0.02; p=0.48).

**Outcome: Comparative Major Complications**

**Figure 23**

**Comments**

- Overall, the Adjuvant group had lower risk than the Neoadjuvant group in developing a major complication and this risk difference was statistically significant i.e. RD = -0.07 (95% CI: -0.14 to -0.01; p=0.04).
- For the Versluis (2023) study, results reported by Blank et al (2018) were used for data extraction i.e. *“However inboth arms, 9/10 patients experienced one or more grade 3/4 adverse events. (see Abstract, page 1655”*

**Outcome: Comparative Patterns of Recurrence**

**Figure 24**

**Comments**

- Overall, the Adjuvant group had a higher risk than the Neoadjuvant group in having a Recurrence and this risk diffrence was statistically significant i.e. RD = 0.18 (95% CI: 0.13 to 0.24; p<0.01).
